# Metagenomic Next-Generation Sequencing Versus Traditional Laboratory Methods for the Diagnosis and Treatment of Infection in Liver Transplantation

**DOI:** 10.3389/fcimb.2022.886359

**Published:** 2022-06-16

**Authors:** Jun-Feng Huang, Qing Miao, Jian-Wen Cheng, Ao Huang, De-Zhen Guo, Ting Wang, Liu-Xiao Yang, Du-Ming Zhu, Ya Cao, Xiao-Wu Huang, Jia Fan, Jian Zhou, Xin-Rong Yang

**Affiliations:** ^1^Liver Surgery Intensive Care Unit, Department of Intensive Care Medicine, Zhongshan Hospital, Fudan University, Shanghai, China; ^2^Department of Liver Surgery and Transplantation, Liver Cancer Institute, Zhongshan Hospital, Fudan University; Key Laboratory of Carcinogenesis and Cancer Invasion (Fudan University), Ministry of Education, Shanghai, China; ^3^Shanghai Key Laboratory of Organ Transplantation, Zhongshan Hospital, Fudan University, Shanghai, China; ^4^Department of Infectious Diseases, Zhongshan Hospital, Fudan University, Shanghai, China; ^5^Cancer Research Institute, Central South University; Key Laboratory of Carcinogenesis and Cancer Invasion, Ministry of Education, Changsha, China; ^6^Institute of Biomedical Sciences, Fudan University, Shanghai, China

**Keywords:** liver transplantation, metagenomic next-generation sequencing, donor-derived infection, perioperative infection, immunocompromised patient

## Abstract

**Background:**

Metagenomic next-generation sequencing (mNGS) has emerged as an effective method for the noninvasive and precise detection of infectious pathogens. However, data are lacking on whether mNGS analyses could be used for the diagnosis and treatment of infection during the perioperative period in patients undergoing liver transplantation (LT).

**Methods:**

From February 2018 to October 2018, we conducted an exploratory study using mNGS and traditional laboratory methods (TMs), including culture, serologic assays, and nucleic acid testing, for pathogen detection in 42 pairs of cadaveric liver donors and their corresponding recipients. Method performance in determining the presence of perioperative infection and guiding subsequent clinical decisions was compared between mNGS and TMs.

**Results:**

The percentage of liver donors with mNGS-positive pathogen results (64.3%, 27/42) was significantly higher than that using TMs (28.6%, 12/42; P<0.05). The percentage of co-infection detected by mNGS in liver donors was 23.8% (10/42) significantly higher than 0.0% (0/42) by TMs (P<0.01). Forty-three pathogens were detected using mNGS, while only 12 pathogens were identified using TMs. The results of the mNGS analyses were consistent with results of the TM analyses in 91.7% (11/12) of donor samples at the species level, while mNGS could be used to detect pathogens in 66.7% (20/30) of donors deemed pathogen-negative using TMs. Identical pathogens were detected in 6 cases of donors and recipients by mNGS, among which 4 cases were finally confirmed as donor-derived infections (DDIs). For TMs, identical pathogens were detected in only 2 cases. Furthermore, 8 recipients developed early symptoms of infection (<7 days) after LT; we adjusted the type of antibiotics and/or discontinued immunosuppressants according to the mNGS results. Of the 8 patients with infections, 7 recipients recovered, and 1 patient died of severe sepsis.

**Conclusions:**

Our preliminary results show that mNGS analyses can provide rapid and precise pathogen detection compared with TMs in a variety of clinical samples from patients undergoing LT. Combined with symptoms of clinical infection, mNGS showed superior advantages over TMs for the early identification and assistance in clinical decision-making for DDIs. mNGS results were critical for the management of perioperative infection in patients undergoing LT.

## Introduction

Liver transplantation (LT) is the most effective treatment for end-stage liver cirrhosis and liver cancer (Dogan and Kutluturk, 2020). The civilian organ donation program has been the sole source of organs for transplant in China since January 2015, and the number of voluntary donations has increased every year (Huang et al., 2015). Infection-related complications have become the leading cause of morbidity and mortality for patients undergoing LT due to the use of immunosuppressive agents (Nam et al., 2018). Perioperative infections are particularly serious and can lead to liver graft failure and even death (Heldman et al., 2019). Such infections in liver recipients can arise from reactivation of latent pathogens, donor-derived infections (DDIs), or primary infections (Pettengill et al., 2019). The early and precise detection of infectious pathogens can be used to optimize the administration of antibiotics and immunosuppressants to improve clinical outcomes for patients undergoing LT (Huang et al., 2020). Therefore, development of a more rapid, sensitive, and specific method for the identification of potential pathogens for these patients is urgently needed.

Traditional laboratory methods (TMs) for the screening of potential pathogens usually include cell culture, serologic assays, and nucleic acid testing. However, testing all potential pathogens in liver donors and corresponding recipients using TMs is extremely time-consuming. Metagenomic next-generation sequencing (mNGS) is a promising approach to determine the presence and abundance of transplant-related infections and identify co-infection in an unbiased manner (Simner et al., 2018). The use of mNGS can overcome the limitations of current diagnostic tests, allowing for hypothesis-free, culture-independent, pathogen detection directly from clinical specimens regardless of the type of microbe; mNGS can even be used for novel organism discovery (Simner et al., 2018). To date, there are few reports on the use of mNGS to identify potential pathogens in liver donors and their corresponding recipients.

In this study, the diagnostic performance of mNGS was evaluated and compared with the use of TMs in patients undergoing LT. Furthermore, the feasibility of using mNGS for the diagnosis and treatment of perioperative infections in LT recipients was evaluated. We found that the use of mNGS provided rapid and precise detection of pathogens compared with TMs in a variety of clinical samples from patients undergoing LT. Combined with symptoms of clinical infection, the use of mNGS could offer an advantage over the use of TMs for the diagnosis of DDIs and the precise treatment of these perioperative infections.

## Materials and Methods

### Ethics Statement

An application for ethical review was approved by the Ethical Review Committee of Zhongshan Hospital affiliated with Fudan University.

### Patients, Perioperative Management, and Sample Collection

This study was a single-center, prospective cohort study from February 1, 2018 to October 30, 2018. A total of 42 cadaveric liver donors and their corresponding recipients were enrolled. All donors’ clinical data were obtained prior to procurement. All recipients received orthotropic LT and induction of immunosuppression intraoperatively with basiliximab and methylprednisolone. The regimen for antibiotic prophylaxis for LT consisted of cefepime and micafungin for 7 days postoperatively. Immunosuppressant therapy after LT consisted of a triple-drug regimen of cyclosporine or tacrolimus, mycophenolate mofetil, and methylprednisolone; the doses of these drugs were decreased over 7 days. Recipient outcomes were examined for the entire length of the hospital stay.

The donor’s samples, including blood, preservation fluid, liver and perihepatic tissue (diaphragm or omentum), were obtained preoperatively. Microbiological monitoring of the liver recipients involved the routine sampling of blood and abdominal drainage fluid on postoperative days (POD) 1, 4, and 7. When the recipient was diagnosed with a postoperative infection, additional samples from the sputum, bronchoalveolar lavage fluid, and urine were collected for pathogen analysis according to the clinical situation. All samples were subjected to TMs as well as mNGS testing in a pairwise manner. TMs for pathogen detection included culture of bacterial and fungal; PCR-based assay of *Epstein-Barr virus* (EBV), *Cytomegalovirus* (CMV), *Hepatitis B virus* (HBV), *Hepatitis C virus* (HCV); serological assay including 1,3-Beta-D-glucan, Galactomannan antigen, Interferon-gamma release assays for *Tuberculosis*, *Cryptococcus* antigen, HBV, HCV and *Human immunodeficiency virus* (HIV) serological test, EBV early antigen and viral capsid antigen, CMV immunoglobulin G/M (IgG/M), *Toxoplasma gondii* IgG/M, Rapid plasma reagin (RPR) test for *Syphilis* and stool microscopy for parasitic ova. The diagnostic assessment performances of the TMs and mNGS were compared.

### Sample Processing

All samples were promptly stored in sterile containers and placed at 4°C prior to analysis. For blood samples, 3–4 mL of blood was centrifuged at 4,000 rpm for 10 min at 4°C within 8 h of collection, and plasma samples were transferred to new sterile tubes. An aliquot of 3–5 mL preservation fluid or drainage fluid was collected, according to standard sterile procedures (Cornaglia et al., 2012). Tissue homogenates, including those of liver and perihepatic tissues, were processed similarly to preservation fluid (Cornaglia et al., 2012); 1.5-mL microcentrifuge tubes containing 0.5 mL sample and 1 g of 0.5-mm glass beads were attached to a horizontal platform on a vortex mixer and agitated vigorously at 2,800-3,200 rpm for 30 min.

### mNGS

DNA was extracted from 300 µL samples using the TIANamp Micro DNA Kit (DP316, TIANGEN BIOTECH, Beijing, China), following the manufacturer’s instructions. DNA libraries were constructed through DNA fragmentation, end-repair, adapter-ligation, and PCR amplification (Long et al., 2016). The reagents were taken out from the kit, and the enzymatic reagents were briefly centrifuged and placed on ice for use; The other reagents were melted on ice, mixed with oscillation, and briefly centrifuged for use. Magnetic beads should be balanced at room temperature for 30min before use, and thoroughly mixed before adding. Anhydrous ethanol and molecular water are used to prepare 75% ethanol. internal standard (200×) was diluted 200 times in nuclease-free water. Then, the terminal repair reaction mixture was prepared for end-repair. The extracted nucleic acid was added, and then the terminal repair reaction mixture 7.0μL was added. The mixture was placed on PCR and incubated. At the end of the reaction, the PCR tube was removed for instantaneous centrifugation. 30.0μL connecting reaction mixture was added to the PCR tube, and the mixture was fully mixed and centrifuged immediately. The mixture was placed on the PCR instrument and incubated for 23 minutes, ligase was used for adapter-ligation. After PCR amplification, Agilent 2100 instrument (Agilent Technologies, Santa Clara, CA) was used for quality control of the DNA libraries and the Qubit 2.0 fluorometer (Invitrogen, Foster City, CA, USA). A qualified double-stranded DNA library was transformed into a single-stranded circular DNA library by DNA denaturation and circularization. DNA nanoballs (DNBs) were generated from single-stranded circular DNA using rolling circle amplification (da Silva et al., 2016). The DNBs were qualified by fluorometry (Fang et al., 2018). Qualified DNBs were loaded in the flow cell and sequenced on the BGISEQ-50 platform (Jeon et al., 2014). High-quality sequencing data were generated by removing low-quality and short reads (length <35 bp), followed by computational subtraction of human host sequences mapped to the human reference genome (hg19) using Burrows-Wheeler alignment (Li and Durbin, 2010). After removal of low-complexity reads, the remaining sequencing data were classified by simultaneous alignment to sequences in the bacterial, viral, fungal, and parasite microbial genome databases.

The reference database RefSeq, downloaded from the National Center Biotechnology Information website (https://ftp.ncbi.nlm.nih.gov/genomes/), contains 4,945 whole-genome sequences of viral taxa, 6,350 bacterial genomes or scaffolds, 1,064 fungal sequences related to human infections, and 234 parasite sequences associated with human diseases.

### Analyses of mNGS Results

The criteria for a positive mNGS result have been described previously (Miao et al., 2018). Briefly, bacteria, viruses, and parasites (species level) were identified with a coverage rate 10-fold greater than that of any other bacteria, virus, or parasite. Fungi (species level) were identified with a coverage rate 5-fold higher than that of any other fungi because of their low biomass after DNA extraction. *Mycobacterium tuberculosis* was considered positive when at least one read was mapped (genus or species level). *Nontuberculous mycobacteria* were considered positive when the mapped read number at either the species or genus level was in the top 10 of the list of bacteria.

The number of unique reads of standardized species (SDSMRN) was defined as the number of reads that were strictly aligned to the genome of a species after normalizing the total number of sequencing reads to 20 million (Li et al., 2020). Probable DDI was defined as the transmission of the identical pathogen detected from donor to recipient by mNGS and/or TMs (Kaul et al., 2021) and the recipient developed early infection symptoms (<7 days) as fever and/or purulent drainage observed with increased markers of laboratory infection after LT (Bandali et al., 2020).

### Statistical Analysis

Sensitivity, specificity, positive predictive value (PPV), negative predictive value (NPV), and accuracy (ACC) were calculated, and the performance of mNGS and TMs for diagnostic assessments was compared using the χ2 test. A two-tailed P value of 0.05 was considered statistically significant. Data were analyzed using SPSS, version 24.0 (SPSS, Chicago, IL, USA).

## Results

### Recipient Characteristics

Demographic features of the recipients in the study are provided in **Table 1**. All 42 recipients underwent orthotopic cadaveric LT. The median patient age was 49 years (range, 21-72 years). Most recipients were male (36/42, 85.7%) and had been diagnosed with primary malignant liver cancer (29/42, 69.1%), followed by decompensated liver cirrhosis (9/42, 21.5%). Ascites was present in 81.0% of recipients (34/42), and antibiotics were used in 33.3% of recipients (14/42) one month before LT for either the treatment of infection or prevention of spontaneous peritonitis due to cirrhosis with ascites or upper gastrointestinal bleeding. Of the 42 patients, 8 recipients were diagnosed with postoperative infection, with pneumonia (6/8, 75.0%) being the most common infection.

**Table 1:** Recipient characteristics (N = 42).

Characteristics	No.	%
Age (years)*		
Median (Range)	49 (21-72)	
**Sex**		
Male	36	85.7
**Outcome**		
Survival	40	95.2
**Hospital stay (days)**		
Median (Range)	21.5 (5-73)	
**Past history**		
Surgery history	19	45.2
Hypertension	7	16.7
Diabetes	6	14.3
**Diagnosis**		
**Malignant tumor of liver**		
Hepatocarcinoma	28	66.7
Intrahepatic cholangiocarcinoma	1	2.4
**Hepatocirrhosis**		
Hepatitis B cirrhosis	7	16.7
Primary biliary cirrhosis	1	2.4
Idiopathic cirrhosis	1	2.4
**Acute liver failure**	1	2.4
**Secondary transplantation**	3	7.1
**Ascites**	34	81.0
**Antibiotic treatment within 1 months before surgery**	14	33.3
**Postoperative infection**	8	19.0
**Postoperative infection site**		
Pneumonia	6	75.0
Sepsis	2	25.0
Urinary tract	3	37.5
Intra-abdominal	4	50.0

*Age at start of liver transplantation surgery.

### The Spectrum of Pathogens in Liver Donors Detected by mNGS and TMs

**Figure 1:**
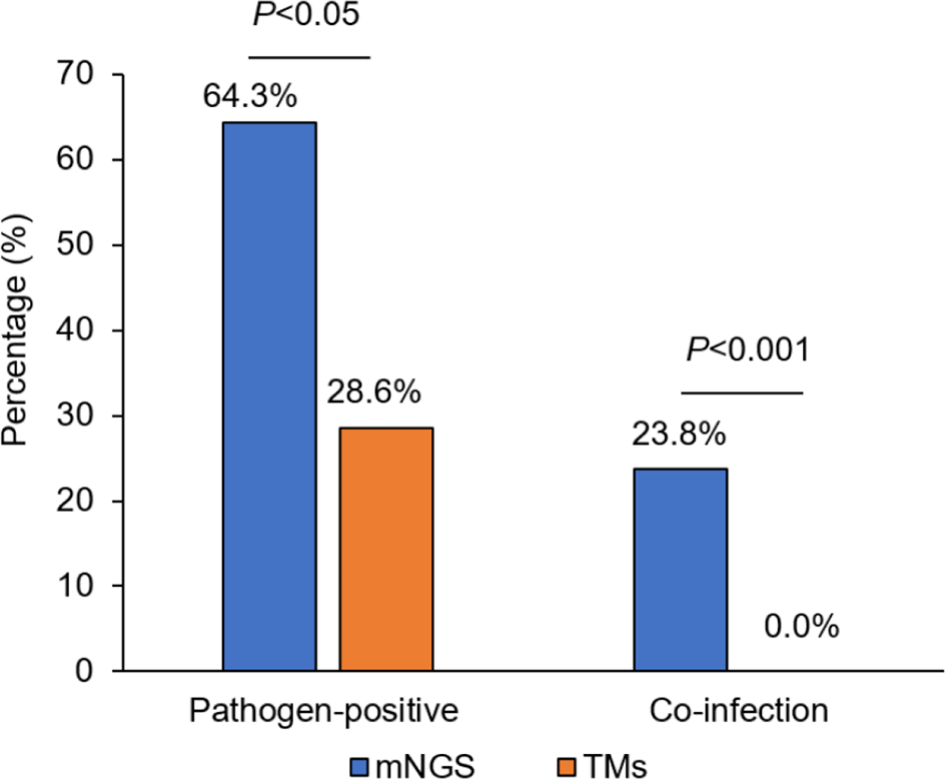
The comparison of pathogen-positive percentage and co-infection rate detected separately by mNGS and TMs in liver donors. mNGS for metagenomic next-generation sequencing and TMs for traditional laboratory methods.

The percentage of liver donors with mNGS-positive pathogen results (64.3%, 27/42) was significantly higher than when using TMs (28.6%, 12/42; P<0.05), and the percentage of co-infection of several common pathogens, detected by mNGS in liver donors was 23.8% (10/42) compared with 0.0% as detected by TMs (0/42; P<0.001; **Figure 1**).

**Figure 2:**
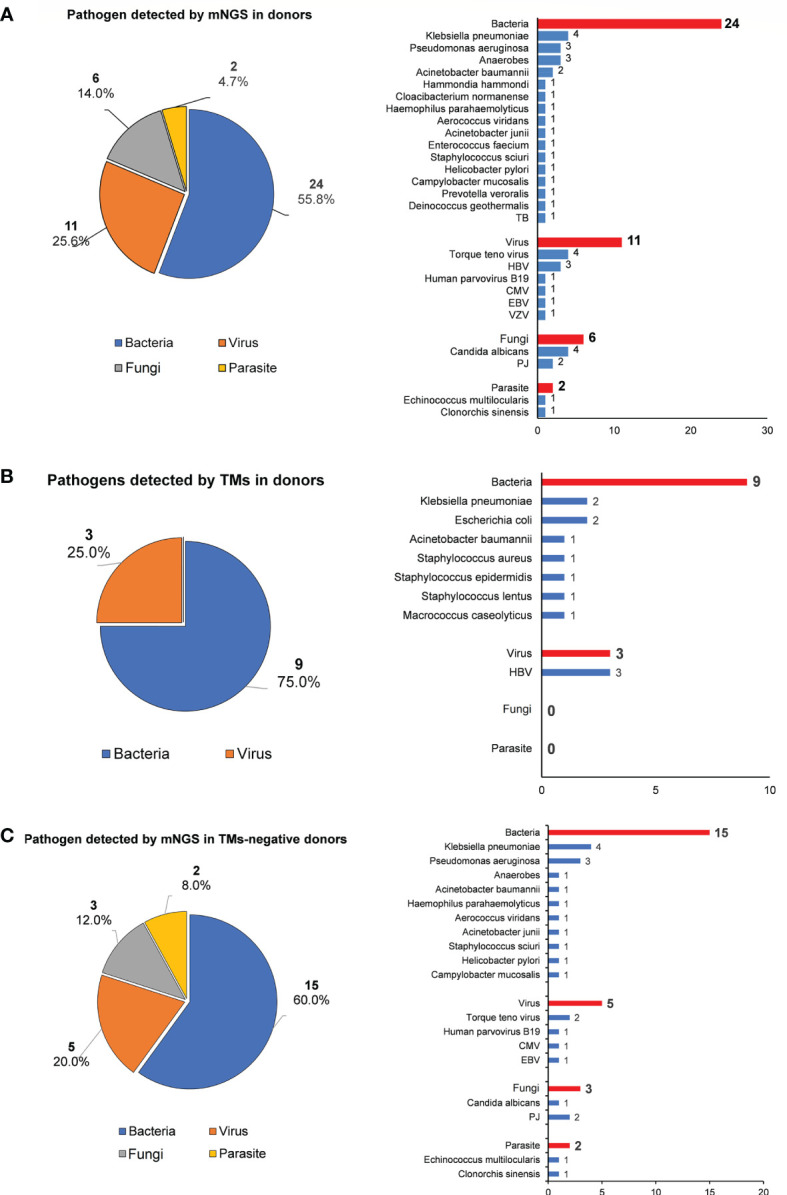
The pathogen spectrum in liver donor detected by mNGS and TMs. **(A)** Pie chart demonstrating the distribution of different types of pathogens detected by NGS in liver donors, and a total of 43 species of pathogens were detected in donor samples with their corresponding frequencies plotted in histograms. **(B)** Pie chare demonstrating the distribution of different types of pathogens detected by TMs in donors, and a total of 12 species of pathogens were detected in donor samples with their corresponding frequencies plotted in histograms. **(C)** Pie chart shows the distribution of different types of pathogens detected in TMs-negative donor samples by mNGS, and species of pathogens were detected with their corresponding frequencies plotted in histograms.

mNGS detected 43 pathogens (bacteria: 55.8%, 24/43; viruses: 25.6%, 11/43; fungi: 14.0%, 6/43; parasites: 4.7%, 2/43), whereas only 12 pathogens were identified using TMs (bacteria: 75.0%, 9/12; viruses: 25.0%, 3/12; **Figures 2A, B**). No fungi or parasites were identified using TMs. Among 30 donors who tested negative for pathogens using TMs, mNGS identified new pathogens in 20 cases (20/30, 66.7%), including fastidious bacteria, fungi [e.g., *Pneumocystis jirovecii* and *Candida albicans* (CA)], virus [e.g., *Torque Teno Virus* (TTV), *Human parvovirus B19*, CMV and EBV] and parasite [e.g., *Echinococcus multilocularis* and *Clonorchis sinensis*]. This result reflected the low sensitivity of TMs in screening donor-derived pathogens (**Figure 2C**).

Among 12 donors who tested positive for pathogens using TMs, the results of the mNGS analysis were consistent in 11 out of 12 (91.7%) donor samples at the species level. However, at least one unique pathogen read was detected by mNGS when co-infection was observed. For the detected pathogen spectrum, common bacteria such as *Klebsiella pneumoniae* (KP), *Acinetobacter baumannii* (AB), *Escherichia coli*, and *Staphylococcus* could be detected by both TMs and mNGS, whereas fungi and unexpected viruses were only able to be identified by mNGS. Inconsistent results were only found for Donor 1 (D1) between mNGS and TMs: *Staphylococcus lentus* was identified by culture in both preservation fluid and perihepatic tissues, but was not detected by mNGS in any D1-related samples (**Table 2**).

**Table 2:** mNGS results of 12 TMs-positive sample of donors.

D	Sample	TMs results	mNGS results				Correlation
			Species	NO. of unique reads	Coverage %	Depth	
1	preservation fluid	Staphylococcus lentus	/	/	/	/	no
	perihepatic tissue	Staphylococcus lentus	/	/	/	/
5	preservation fluid	Escherichia coli	Bacteroides vulgatus	50	0.0978	1	yes
			Corynebacterium urealyticum	32	0.1191	1
			Escherichia coli	1	0.0009	1
14	perihepatic tissue	Staphylococcus aureus	Staphylococcus aureus	10	0.0889	1	yes
17^#^	blood/liver tissue	HBV	HBV	13	39.25	1.33	yes
19	preservation fluid	Macrococcus caseolyticus	Macrococcus caseolyticus	122	0.6779	1.02	yes
			Torque teno mini virus 7	1	3.46	1
			Torque teno virus 15	1	7.84	1
24*	preservation fluid	Klebsiella pneumoniae	Klebsiella pneumoniae	63	0.0565	1	yes
			Candida albicans	7	0.0031	1
			Human herpesvirus 3	4	0.3195	1
28*	preservation fluid	Klebsiella pneumoniae	Klebsiella pneumoniae	898	1.98	1.02	yes
			Prevotella veroralis	882	2.03	1.02
			Candida albicans	15	0.0067	1
	perihepatic tissue	Klebsiella pneumoniae	Klebsiella pneumoniae	1657	3.57	1.03
			Prevotella veroralis	1307	3.05	1.03
			Candida albicans	15	0.0056	1
29^#^	blood/liver tissue	HBV	HBV	6	21.74	1.17	yes
32^#^	blood/liver tissue	HBV	HBV	2	4.67	1	yes
36	preservation fluid	Staphylococcus epidermidis	Staphylococcus epidermidis	14	0.0381	1	yes
	perihepatic tissue	Staphylococcus epidermidis	Staphylococcus epidermidis	1	0.0019	1
39	preservation fluid	Escherichia coli	Escherichia coli	1	0.0101	1	yes
41*	perihepatic tissue	Acinetobacter baumannii	Acinetobacter baumannii	32	0.0448	1	yes
			Klebsiella pneumoniae	21	0.035	1
			Candida albicans	4	0.0014	1

D, Donor; TMs, Traditional laboratory methods; mNGS, Metagenomic next-generation sequencing; HBV, Hepatitis B Virus; ^#^donor was HBV positive; *Donor derived infection.

### The Performance of TMs and mNGS for the Detection of Donor-Recipient Transmitted Pathogens

The performance of TMs and mNGS for the detection of donor-transmitted pathogens was further evaluated. The transmission of pathogens was detected in 6 cases (6/42) from donor to recipient by mNGS, among which 4 cases were confirmed as DDIs with a 100% sensitivity and a 94.7% specificity. By TMs, the transmission was only detected in 2 cases (2/42) confirmed as DDI with sensitivity of 50.0% and a specificity of 100%. The PPV and NPV of mNGS were 66.7% and 100% for DDI diagnoses, respectively, compared with 100.0% and 95.0% for TMs, respectively. The ACC of both mNGS and TMs was 95.2% (**Table 3**). The new emerging pathogens transmitted *via* liver graft were identified by mNGS in six cases (**Table 4**). We found that KP (3/6, 50.0%), AB (2/6, 33.3%), *Varicella zoster virus* (VZV) (1/6, 16.7%), *Candida glabrata* (CG) (1/6, 16.7%), and *Clonorchis sinensis* (CS) (1/6, 16.7%) were detected by mNGS in both donor and corresponding recipient. There were 2 cases (2/6, 33.3%) with co-infection detected by mNGS in samples from D24 and D41, which could not be identified by TMs, especially the fungi and viruses.

**Table 3:** Comparison of sensitivity and specificity between NGS and TMs in diagnosis of DDI.

		DDI	Non-DDI	Sensitivity	Specificity	PPV	NPV	ACC
**NGS**	**positive**	4	2	100.0%	94.7%	66.7%	100.0%	95.2%
	**negative**	0	36					
**TMs**	**positive**	2	0	50.0%	100.0%	100.0%	95.0%	95.2%
	**negative**	2	38					

DDI, Donor derived infection; PPV, Positive predictive value; NPV, Negative predictive value; ACC, Accuracy.

**Table 4:** Transmission of pathogens detected by mNGS and TMs from liver donor to corresponding recipient.

mNGS										TMs				
ID	Donor				Recipient					Donor			RecipientPOD 1	
					Pre-LT	POD 1								
	blood	pre- servation fluid	peri- hepatic tissue	liver tissue	blood	blood	drainage			blood	pre- servation fluid	peri- hepatic tissue	blood	drainage fluid
							right	middle	left					
**3**		CS	CS	CS	BKPyV		AB CS		CS					
24*		VZV	VZV			KP	VZV	VZV	VZV		KP		KP	
		KP												
28*	KP	KP	KP	KP	HBV TTV	KP	KP				KP	KP	KP	
**35**		KP	KP	KP	AF		KP	KP	KP					
41*	AB		AB	AB	TTV	AB	AB	AB	AB		AB			
	KP		KP	KP		KP	KP	KP	TTV					
	CG		CG	CG		CG	CG	CG						
							TTV							
42*		AB				AB	AB	AB	AB					

AB, Acinetobacter baumannii; AF, Aspergillus flavus; BKPyV, BK polyomavirus; CG, Candida glabrata; CS, Clonorchis sinensis; HBV, Hepatitis B virus; KP, Klebsiella pneumoniae;

LT, Liver transplantation; POD, Post operation day; TTV, Torque teno virus; VZV, Varicella zoster virus.

*Donor derived infection.

### Guided Treatment for Perioperative Infection by mNGS in Patients When TMs Results Appear Invalid

The postoperative treatment regimen of antibiotics and immunosuppressants are first routinely adjusted according to the pathogens detected in donor and corresponding recipient by TMs. Under this situation, a total of 8/42 (19.0%) recipients developed early infection symptoms (<7 days) (**Table 5**). The mean time was 2.0 days to symptom onset (range, 1–6 days) after LT, and clinical manifestations included fever, purulent drainage fluid, increased procalcitonin (PCT) or C-reactive protein (CRP) levels, and positive imaging results. Sequentially, we modified the therapeutic regimen according to the results of mNGS in all eight infected recipients, and finally seven recipients recovered and one (R24) died of severe sepsis.

**Table 5:** The precise treatment of perioperative infection guided by mNGS infailure cases of TMs in LT.

R	The time of symptoms onset	TMs based diagnosis		mNGS based diagnosis		Changes in treatment strategies by mNGS results		Follow-up results
3	POD1	Negative		AB intra-abdominal infection		Cefepime changed to tigecycline		Recovery
7	POD1	AB and Candida glabrata pneumonia		CMV and TTV detected in blood and drainage		Added ganciclovir		Recovery
24	POD1	Sepsis and pneumonia (donor derived CRKP infection)		Probable DDI. KP bloodstream infection and pneumonia; VZV detected in donor and subsequently in abdominal drainage of recipient		Added ganciclovir and withdraw immunosuppressant		Death on POD 6
28	POD1	Sepsis, abdominal infection and pneumonia (donor derived CRKP infection)		Probable DDI. KP bloodstream infection, intra-abdominal infection and pneumonia		Withdraw immunosuppressant; dynamic changes in reads guided the course of antibiotics use, while blood culture result had been negative		Recovery
39	POD6	Urinary tract Enterococcus faecium and SM infection		Both blood and abdominal drainage detected negative		Meropenem changed to cefoperazone sulbactam, and then was discontinued		Recovery
40	POD1	Candida tropicalis pneunomia		Aspergillus fumigatus and candida tropicalis detected in BALF		Added voriconazole		Recovery
41	POD1	AB abdominal infection; (AB was positive in culture of preservation fluid)		Probable DDI; AB, KP and CA simultaneously detected in donor as well as in blood and abdominal drainage of recipient		Added polymyxin B for bloodstream infection and withdraw immunosuppressant		Recovery
42	POD4	Candida tropicalis and SM cultured positively in sputum		Probable DDI; AB simultaneously detected in donor and subsequently in abdominal drainage and blood of recipient		Added tigecycline and withdraw immunosuppressant		Recovery

R, recipient; TMs, Traditional laboratory methods; LT, liver transplant; POD, Post operation day; AB, Acinetobacter baumannii; CMV, Cytomegalovirus; TTV, Torque teno virus; CRKP, Carbapenem resistant klebsiella pneumoniae; DDI, Donor derived infection; KP, Klebsiella pneumoniae; CA, Candida albicans; VZV, Varicella zoster virus; SM, Stenotrophomonas maltophilia; BALF, Bronchoalveolar lavage fluid.

Types of antibiotics were adjusted and/or immunosuppressants were withdrawed for R3, R7, R24, R28, R40, R41, and R42 (7/8, 87.5%), according to the additional pathogens identified by mNGS; the mNGS-negative results in R39 led to discontinue unnecessary broad-spectrum antibiotics (such as meropenem, which was replaced by cefoperazone sulbactam and then discontinued) (**Table 5**). The dynamic changes in SDSMRN by continuous mNGS surveillance in R28 guided the complete course of antibiotics for 30 days, as result of blood culture had been negative from POD 11 (**Table 6**). One recipient (R24) died after treatment adjustment and had received LT for drug-related acute liver failure (Model for End-stage Liver Disease score 41). In this case, VZV was only detected by mNGS in the donor liver and subsequently found in the abdominal drainage fluid of the recipient, suggesting a latent VZV infection in the donor. Despite the prompt use of antiviral therapy guided by mNGS, the recipient still succumbed to severe sepsis, which resulted in fatal liver failure on POD 6.

**Table 6:** Dynamic changes in standardized unique read of CRKP by mNGS.

	POD 1	4	7	11	14	21	30
**SDSMRN**	121	324	537	390	246	177	9
**Blood culture**	CRKP	CRKP	CRKP	N	N	N	/

POD, Post operation day; SDSMRN, The number of unique reads of standardized species; CRKP, Carbapenem resistant klebsiella pneumoniae; N, Negative.

## Discussion

Infection-related complications have become the leading cause of morbidity and mortality for patients in the first months after LT (Nam et al., 2018). The early and precise detection of infectious pathogens can optimize the use of targeted antibiotics and immunosuppressants to improve clinical outcomes for patients after LT. It is great challenge to test all potential pathogens existing in liver donors and their corresponding recipients using TMs. mNGS can overcome the limitations of the current diagnostic testing (Zhou et al., 2019). In this study, we prospectively evaluated the clinical value of mNGS for the identification of pathogens in different types of samples during LT. We found that mNGS could provide rapid and precise detection of pathogens and was an ideal tool for the diagnosis of DDIs. Furthermore, mNGS was able to guide the precise treatment of perioperative infections in patients undergoing LT.

Our data revealed that positive results using mNGS analyses were consistent with those from TMs 91.7% of the time. These results suggest that mNGS can effectively detect the same pathogens as TMs and identify more latent pathogens carried by donors. As mNGS analyses often detected more than one pathogen in a single test, clinicians need to have a comprehensive understanding of results indicating the presence of co-infection (Tarabichi et al., 2018). Thus, we used our own criteria (Miao et al., 2018) to uncover co-infections and/or distinguish the causative pathogens. We found that CA (4/43, 9.3%) was the most common fungi within the co-infection detected by mNGS. Donor-derived fungal infections have been associated with life-threatening complications in transplant recipients (Mishkin, 2021), so mNGS analyses would allow the precise and timely detection of fungi to enable prompt treatment. It is worth noting that the average turnaround time for culture results is more than 72 hours for commonly encountered bacteria and up to weeks for more insidious pathogens such as *Aspergillus fumigates* (Simner et al., 2018). Thus, the turnaround time of mNGS (average of 48 hours) (Pendleton et al., 2017; Afshinnekoo et al., 2017) will hasten clinical decision-making, which is critical for immunocompromised recipients after LT (Simner et al., 2018).

Metagenomic sequencing combined with phylogenetic analysis could effectively identify the frequent transmission of* JC polyomavirus* from kidney transplant donor to recipient (Schreiber et al., 2019). Our study demonstrated the transmission of identical pathogens from donors to corresponding recipients in 6 of 42 cases (14.3%) by mNGS, which can promptly assist in the diagnosis of DDIs with clinical infection symptoms. As compared with TMs, mNGS was more sensitive (100% vs. 50%) with a similar specificity (94.7% vs. 100.0%) in terms of diagnoses of DDIs, respectively. As immunocompromised recipients are generally critical ill, the timely identification of the pathogens causing DDI is crucial for a precise diagnosis, which is necessary for proper treatment (Nam et al., 2018). More importantly, the mNGS-negative results of donor and corresponding recipient can assist to exclude DDI in clinical work.

With antimicrobial treatment guided by the TMs results, 8 of 42 (19.0%) recipients developed an early infection (<7 days) after LT. Targeted antibiotics were adjusted and/or immunosuppressants were discontinued according to the additional pathogens identified by mNGS. Finally, 7 recipients recovered. These examples demonstrate that mNGS can effectively guide the treatment of perioperative infection after LT, especially when routine TMs results appear inefficacy. The decrease or disappear of pathogen unique reads monitored by mNGS are correlated with improvement of clinical infection symptoms and indirectly guide the course of antibiotics (Zhang et al., 2019), especially in DDI cases. Blood culture of one recipient (R28) with DDI had been negative since POD 11, the course of antibiotics was guided by dynamic changes of unique pathogen reads detected by mNGS with clinical index of PCT for 30 days. Therefore, mNGS results might be a reliable indicator to help understand how the pathogens progress and guide the adjustment of antibiotics in DDI cases (Ai et al., 2018).

TTV load is modulated by the immune, viral, and inflammatory status, and often considered as potential marker associated with immunity status as well as infectious diseases in LT (Mrzljak and Vilibic-Cavlek, 2020). As reported, TTV viremia was significantly higher during CMV infections (Ruiz et al., 2019). In our cohort, CMV and TTV were detected in blood and drainage in one recipient (R7) with early infection (<7 days) (**Table 5**), but we did not find TTV in the other infection recipients. The reason might be TTV loads progressively increased and peaks around 3 months post-transplant, positively correlating with the intensity of immunosuppression (Schreiber et al., 2019; Mrzljak and Vilibic-Cavlek, 2020), and then virus specific PCR monitoring will have higher sensitivity for detection of TTV loads after LT.

There was a discordant result between TMs and mNGS in one donor (D1). *S. lentus* was positively identified by TMs in preservation fluid and perihepatic tissue, but was not detected by mNGS. A possible reason may be that pathogen reads make up a minute fraction of the sequencing results and are of low sequencing depth, which means mNGS results could be further improved by increasing sequencing depth. One recipient (R24) died of fulminant liver failure and severe sepsis even with treatment guided by mNGS results. It is worth noting that this outcome might be associated with the poor condition of the recipient prior to LT and acute liver graft dysfunction caused by the recurrence of VZV infection after LT. Disseminated visceral VZV infection has been described as a rare but severe disease with a high mortality rate, especially in immunocompromised hosts (Kikuchi et al., 2019). More early diagnosis and timely intervention for those patients, such as the discontinuation of immunosuppressants, might be crucial to improve their clinical outcome (Mehta et al., 2021).

There were some limitations of our study. First, the results of the mNGS were not reconfirmed by PCR-based assays, and a phylogenetic analysis of the pathogens from both the donor and corresponding recipient will be very helpful to confirm diagnosis of DDI. Second, there lacks a unified standardized protocol for mNGS currently in clinical diagnosis. Due to potential breadth of detection and nucleic acid contamination in the process, interpretation of mNGS results directly from clinical specimens can be difficult and requires careful consideration. Additionally, unbiased mNGS was not routinely performed alongside RNA sequencing.

untargeted mNGS was not routinely performed alongside RNA sequencing.

Our study showed that, as compared with TMs, mNGS could yield higher sensitivity for the early identification of fastidious pathogens in patients undergoing LT, especially for DDI diagnoses. Importantly, mNGS does not replace current TMs. Alternatively, it may be considered for immunocompromised patients where achieving a timely diagnosis and treatment is imperative for improved outcomes. The large-scale multicenter randomized controlled studies are needed to further confirm the value of mNGS in routine clinical care of patients undergoing LT.

## Data Availability Statement

The data presented in the study are deposited in the CNGB Sequence Archive (CNSA) of China National GeneBank DataBase (CNGBdb), accession number CNP0003068. (http://db.cngb.org/cnsa/project/CNP0003068_275abe06/reviewlink/).

## Ethics Statement

The studies involving human participants were reviewed and approved by the Ethical Review Committee of Zhongshan Hospital affiliated with Fudan University. The patients/participants provided their written informed consent to participate in this study.

## Author Contributions

Research design was conceived by X-RY, JZ and J-FH. J-FH, QM and J-WC performed the research and analyzed the results. First draft manuscript was prepared by J-FH and QM. AH, D-ZG, TW, L-XY, D-MZ, YC, X-WH and JF contributed to manuscript revisions. All authors contributed to the article and approved the submitted version.

## Funding

This study was jointly supported by the National Key R&D Program of China (2019YFC1315800, 2019YFC1315802), the State Key Program of National Natural Science of China (81830102), the National Natural Science Foundation of China (82150004, 81772578, 81772551, 81872355 and 82072715), the Shanghai Municipal Health Commission Collaborative Innovation Cluster Project (2019CXJQ02), Shanghai “Rising Stars of Medical Talent” Youth Development Program (Outstanding Youth Medical Talents), the Projects from the Shanghai Science and Technology Commission (19441905000 and 21140900300), Shanghai Municipal Key Clinical Specialty.

## Conflict of Interest

The authors declare that the research was conducted in the absence of any commercial or financial relationships that could be construed as a potential conflict of interest.

The reviewer CZ declared a shared parent affiliation with the authors J-FH, QM, J-WC, AH, D-ZG, TW, L-XY, D-MZ, X-WH, JF, JZ, X-RY to the handling editor at the time of review.

## Publisher’s Note

All claims expressed in this article are solely those of the authors and do not necessarily represent those of their affiliated organizations, or those of the publisher, the editors and the reviewers. Any product that may be evaluated in this article, or claim that may be made by its manufacturer, is not guaranteed or endorsed by the publisher.

## Abbreviations

AB, acinetobacter baumannii; ACC, accuracy; CA, candida albicans; CMV, Cytomegalovirus; CRP, C-reactive protein; CS, clonorchis sinensis; D, donor; DDI, donor-derived infection; DNBs, DNA nanoballs; EBV, Epstein-Barr virus; HBV, Hepatitis B virus; HCV, Hepatitis C virus; HIV, Human immunodeficiency virus; KP, klebsiella pneumoniae; LT, liver transplantation; mNGS, metagenomic next-generation sequencing; NPV, negative predictive value; PCT, procalcitonin; POD, postoperative days; PPV, positive predictive value; R, recipient; RPR, Rapid plasma reagin; SDSMRN, the number of unique reads of standardized species; TMs, traditional laboratory methods; TTV, Torque Teno Virus; VZV, varicella zoster virus.
